# Novel approach in whole genome mining and transcriptome analysis reveal conserved RiPPs in *Trichoderma* spp

**DOI:** 10.1186/s12864-020-6653-6

**Published:** 2020-03-27

**Authors:** Gabriel A. Vignolle, Robert L. Mach, Astrid R. Mach-Aigner, Christian Derntl

**Affiliations:** Institute of Chemical, Environmental and Bioscience Engineering, TU Wien, Gumpendorfer Strasse 1a, 1060 Wien, Austria

**Keywords:** Genome mining, RiPP, *Trichoderma*, Ascomycota, Basidiomycota, Secondary metabolism

## Abstract

**Background:**

Ribosomally synthesized and post-translationally modified peptides (RiPPs) are a highly diverse group of secondary metabolites (SM) of bacterial and fungal origin. While RiPPs have been intensively studied in bacteria, little is known about fungal RiPPs. In Fungi only six classes of RiPPs are described. Current strategies for genome mining are based on these six known classes. However, the genes involved in the biosynthesis of theses RiPPs are normally organized in biosynthetic gene clusters (BGC) in fungi.

**Results:**

Here we describe a comprehensive strategy to mine fungal genomes for RiPPs by combining and adapting existing tools (e.g. antiSMASH and RiPPMiner) followed by extensive manual curation based on conserved domain identification, (comparative) phylogenetic analysis, and RNASeq data. Deploying this strategy, we could successfully rediscover already known fungal RiPPs. Further, we analysed four fungal genomes from the *Trichoderma* genus. We were able to find novel potential RiPP BGCs in *Trichoderma* using our unconventional mining approach.

**Conclusion:**

We demonstrate that the unusual mining approach using tools developed for bacteria can be used in fungi, when carefully curated. Our study is the first report of the potential of *Trichoderma* to produce RiPPs, the detected clusters encode novel uncharacterized RiPPs. The method described in our study will lead to further mining efforts in all subdivisions of the fungal kingdom.

## Background

Secondary metabolites (SMs) from fungal sources have played a crucial role in improving human health not only since the discovery of Penicillin, but even in prehistoric times [1, 2]. These natural products and chemically modified variants are widely used as antibiotics, immunomodulators and anti-cancer drugs [3]. Generally well-known examples of fungal SMs belong to two main classes. They are either polyketides (e.g. the mycotoxin aflatoxin and the cholesterol-lowering drug lovastatin) or non-ribosomal peptides (e.g. the antibiotic penicillin and the immunosuppressant cyclosporine). However, also other SM classes are present in fungi: e.g. terpenes, melanins [4, 5], and ribosomally synthesized and post-translationally modified peptides (RiPPs). RiPPs are a rapid growing group of natural products that can be classified in more than 20 different compound classes. Please refer to the reviews by Arnison, P. G. et al. and Luo, S. & Dong, S. H [6, 7]. Small peptides are of increasing interest due to unique bioactive properties aiming at “undruggable” diseases and successfully eradicating anti-biotic resistant microorganisms [8]. The many applications of natural cyclic peptides, including potent lipid-lowering effects of fungal cyclic peptides, are reviewed by Abdalla, M. A. & McGaw, L. J [9].

It is important to differentiate RiPPs from fungal Kexin-like proteinase (KEX2)-processed repeat proteins called KEPs. KEPs are small secreted peptides that do not undergo post-translational modifications, their precursor peptide is cleaved by different proteases and then released by exocytosis [10]. As described by Le Marquer et al., many of these KEPs are putative sexual pheromones but may also play other important roles.

**Fig. 1:**
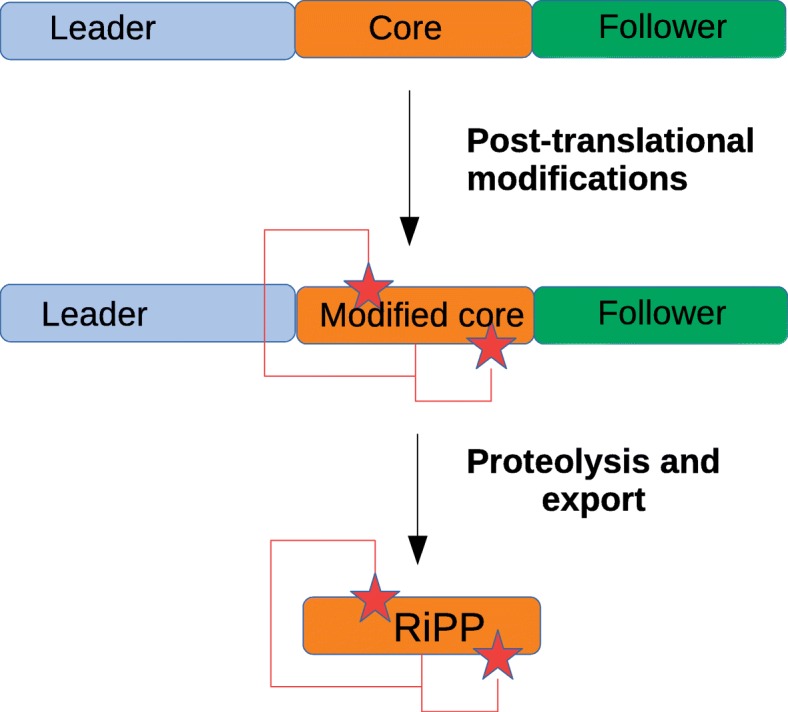
General RiPP biosynthetic pathway. The leader and follower peptide direct the modifications (e.g. addition of functional groups, indicated by stars, or formation of additional bonds, indicated by the connective lines) on the core peptide. After removal of the leader and follower sequence the mature RiPP is released. The figure is an adaptation of the original figure in [6]

Biosynthesis of RiPPs follows a very straight forward production pathway (Fig. 1). A precursor peptide consisting of a leader, a core and a follower amino acid sequence is synthesized by the ribosome. The subsequent post-translational modifications of the core sequence are mediated by modifying enzymes as specified by the leader and follower sequences. After removal of the leader and the follower sequences, the finished bioactive RiPP is released. Many RiPPs undergo a cyclisation step that stabilizes them, reduces their toxicity, improves binding affinity and selectivity. These properties make cyclized RiPPs very attractive candidates for drug development. This labels fungal RiPPs the potential next generation therapeutics [11]. However, only six different classes of RiPPs are described in fungi, yet. Two classes are found in basidiomycetes, i.e. the amatoxins and phallotoxins in the genus *Amanita,* and the borosins with selective nematotoxic activity in *Omphalotus olearius*. RiPPs produced by ascomycetes are the dikaritins and are classified as ustiloxins, asperipins, phomopsins and epichloëcyclins [7].

The genes encoding for the biosynthetic enzymes for SMs are often arranged in individual clusters named biosynthetic gene cluster (BGC), regardless of the class of SM [1]. This organization of clusters is also given for the previously described fungal RiPPs ustiloxins, phomopsins, amatoxins, phallotoxins, borosins and asperipins [7]. This clustered organization is one important feature for the in silico identification of BGCs. Recent advances in next generation sequencing (NGS) lead to the publication of more and more high-quality full genomes from various fungal species and genera such as *Aspergillus flavus* or various *Trichoderma* spp. [12, 13]. Today, fungi represent a vast and generally untapped pool for new lead compounds with pharmaceutical and agricultural applications [14]. However, efforts in genome mining for the search of RiPP BGCs, that encode for the machinery responsible to produce secondary metabolites, have thus far been focused on bacterial genomes due to the lack of a large database of fungal RiPPs [11, 15]. Therefore, most bioinformatic tools available are tailored to mine bacterial genomes for RiPPs.

The current online version of antiSMASH ver. 5.0 includes the identification of RiPP clusters in fungal genomes based on the query sequence (YVIPID) of the putative precursor peptide sequence of phomopsin and *ustYa/ustYb* together with the *ustA* precursor peptide of the ustiloxin cluster [16]. This approach, although being restrictive in its potential to detect novel classes, will aid in the mining for ustiloxin and phomopsin like RiPPs in fungi. Previously, this approach was able to detect 94 putative RiPP precursor peptides in *Aspergillus* spp. This led to the discovery of structurally new cyclic peptides (Asperipins) even though the clusters exhibit high homology to the ustiloxin clusters [7, 17, 18]. We reason that a broader, unconventional forward approach for the detection of putative precursor peptides can be achieved by utilisation and adaptation of bioinformatic tools developed for bacteria. This approach might lead to the discovery of novel fungal RiPPs with potentially new applications and unknown adjacent modifying enzymes. These novel enzymes and the identified precursor peptides can furthermore be used to identify more homologous RiPP BGCs across the fungal kingdom as it was done for the ustiloxin cluster, thereby broadening our search parameters for novel RiPP BGCs.

*Trichoderma* spp. are mesophilic ascomycetes and part of the sordariomycetes, one of the largest classes within their division. The genus *Trichoderma* contains mycoparasitic, saprophytic and opportunistically pathogenic fungi. *T. reesei* is a well-studied saprobe and used industrially for the production of cellulases and hemicellulases [12]. *T. harzianum* is a ubiquitous species with agricultural applications, the opportunistically pathogenic *T. citrinoviride* is often isolated as endophyte and *T. brevicompactum* is a producer of antifungal metabolites [12, 19–21]. All mentioned *Trichoderma* species contain various classes of BGCs, Type 1 polyketide synthetases (T1pks), nonribosomal peptide synthases (NRPSs), terpene BGCs, fatty acid BGCs and various combined and putative clusters.

In this study we demonstrate in silico that by combining antiSMASH [22], the ClusterFinder algorithm and a full HMMer analysis a large set of putative SM BGCs can be identified. After cross-referencing the individual results, we predicted potential RiPP precursor peptides. These sequences were further refined by using previously published RNASeq data [23] and thereby providing a comprehensive highly probable in silico prediction backed up with genomic and transcriptional data.

## Results

### Diversity of secondary metabolite gene clusters in *Trichoderma* spp. and known fungal RiPP producers

First, we compared the biosynthetic gene clusters diversity of nine randomly chosen *Trichoderma* species for which high quality genomes were available. To this end, they were all mined with the command line version of antiSMASH ver. 4.3.0 [22]. We also mined the genomes of *A. flavus* and *Amanita phalloides* in which fungal RiPPs were previously described. The results of the mining with the command line version of antiSMASH are shown in Table 1. The total number of SM BGCs ranges from 11 for the *A. phalloides* genome to 186 found in the SM producer *A. flavus*. There was neither Type 3 pks clusters found in the *Trichoderma* spp. nor any siderophore or indole clusters. Notably, antiSMASH ver. 4.3.0 [22] does not yet include the search for fungal RiPP clusters. The web based antiSMASH ver. 5.0 [16] contains this feature, and was able to detect the ustiloxin B cluster in the *A. flavus* genome, but no other fungal RiPP clusters were found in the mined genomes. Nevertheless, the *Trichoderma* spp. already display a high potential to produce a diverse range of SMs, based on the antiSMASH results.

**Table 1:** Prediction of SM BCGs using antiSMASH ver. 4.3.0

Species	Total BGC	NRPS	T1pks	T3pks	Sid.^**a**^	Ter^**b**^	Ind^**c**^	Mix^**d**^	Other	fatty acid	putativ
*T. reesei*	80	7	9	0	0	6	0	3	4	1	50
*T. citrinoviride*	85	8	8	0	0	5	0	4	6	2	52
*T. harzianum*	129	5	19	0	0	7	0	8	8	2	80
*T. brevicompactum*	96	9	14	0	0	5	0	6	6	2	54
*T. asperellum*	92	5	9	0	0	7	0	6	4	2	59
*T. arundinaceum*	117	9	14	0	0	8	0	11	7	2	66
*T. atrobrunneum*	114	9	18	0	0	5	0	8	7	2	65
*T. koningii*	69	7	9	0	0	4	0	2	4	2	41
*T. virens*	145	16	14	0	0	10	0	8	11	2	84
*A. flavus*	186	11	19	2	1	10	4	10	12	3	114
*A. phalloides*	11	0	1	0	0	5	0	0	1	1	3

^a^Siderophore, ^b^Terpene, ^c^Indole, ^d^T1pks-NRPS/Mix

**Fig. 2:**
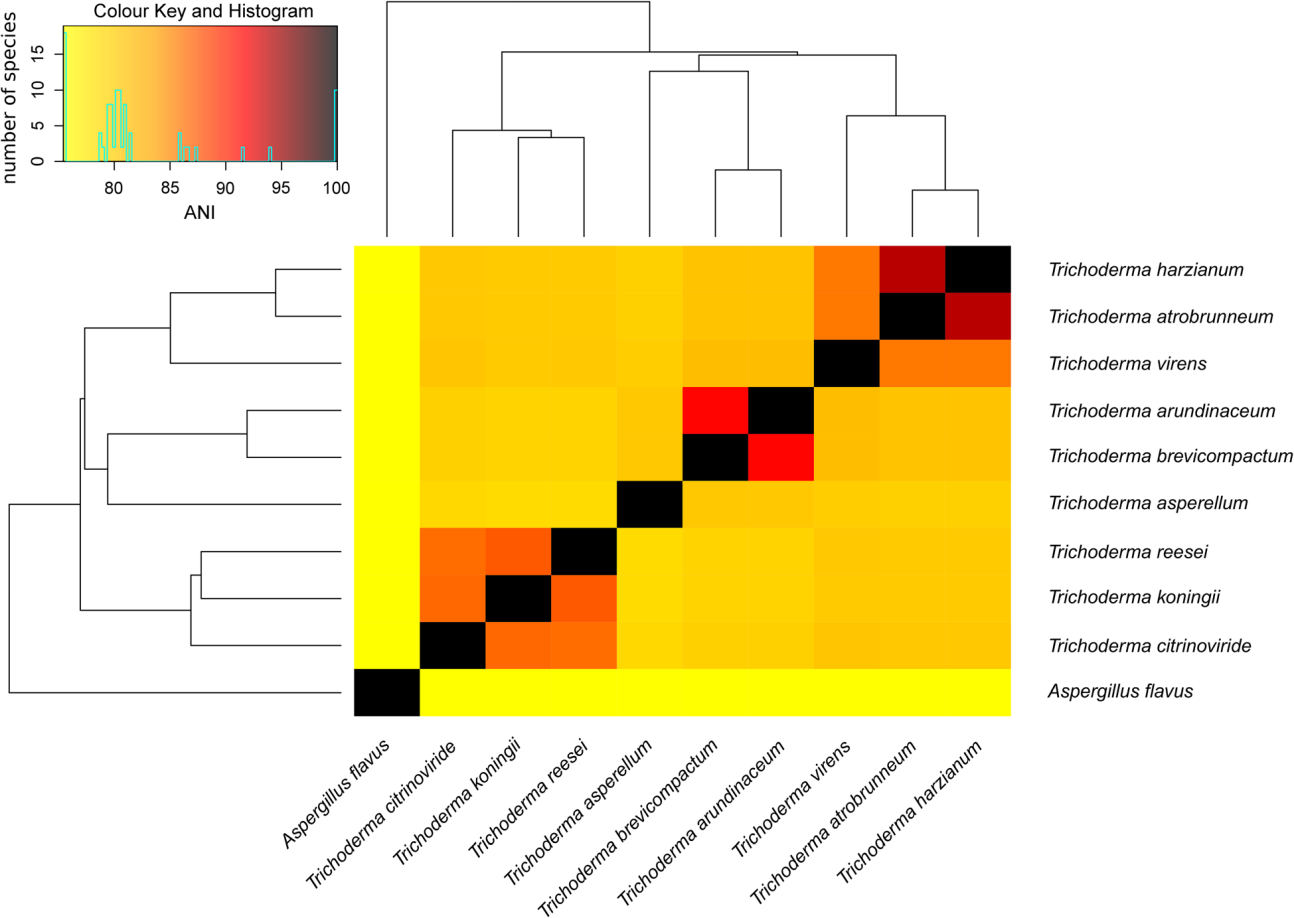
Heatmap of the average nucleotide identity (ANI) between the analyzed fungal species. The respective ANI value is represented by the color gradient. In addition, a histogram indicates the number of species with that certain ANI value. The dendrogram in the heatmap is computed with the complete linkage method to find similar clusters based on the Euclidean distance, representing a whole genome phylogeny

Next, we calculated the average nucleotide identity (ANI) for each strain against each other (Fig. 2). Within the *Trichoderma* spp. there are three distinct clusters detectable based on the ANI value and the computed dendrogram when applying 85% ANI as cutoff. The first containing *T. harzianum*, *T. atrobrunneum* and *T. virens;* the second *T. arundinaceum* and *T. brevicompactum*; the third *T. reesei*, *T. koningii* and *T. citrinoviride*. Based on these findings *T. reesei* and one high quality genome from each cluster were chosen to be mined for putative RiPP precursor genes namely *T. harzianum*, *T. citrinoviride* and *T. brevicompactum*.

### The RiPPMiner standalone tool detects fungal RiPP precursors

As a prerequisite for our analysis, we needed to test the applicability of the RiPPMiner [24] software to recognize precursor peptides of fungal RiPPs. To this end, we tested the software on known precursor peptides of fungal RiPPs extracted from the UniProt database, namely precursors for α-amanitin (A8W7M4), β-amanitin (ABW87785), phallacidin (ABW87771), phalloidin (ABW87787) and 75 diverse known precursor peptides (see Additional file 1). RiPPMiner was able to recognize all of them as precursor peptides, even though it classified them into bacterial groups and predicted improper structure models (Additional file 1). This is a consequence of the used model for these predictions; the model is based on a manually curated database of known precursor peptides of bacterial RiPPs. The precursor peptides for α-amanitin and β-amanitin were predicted to be lassopeptides, whereas phallacidin and phalloidin had no RiPP class prediction (Additional file 1). Next, we evaluated the potential of the RiPPMiner to detect RiPP precursors in known RiPP BGCs and distinguish them from functional polypeptides. To this end, we extracted the sequences from the ustiloxin B BGC from *A. flavus* using the web based antiSMASH ver. 5.0 [16] output and subjected them to an analysis using the RiPPMiner software (Additional file 2). The RiPPMiner was able to detect the verified RiPP precursor (#INPUT 13, AFLA_095020) but misclassified it as cyanobactin. The RiPPMiner predicted three additional input sequences (AFLA_094930, AFLA_094970, AFLA_095000) as RiPP precursors (Additional File 2). These are hypothetical proteins without predicted conserved regions [17]. These results demonstrate that the RiPPMiner software is able to identify fungal RiPP precursors, although it was designed to predict bacterial RiPPs. This leads to misclassifications and false positives.

### Genome mining of *Trichoderma* spp. for putative RiPP precursors

**Fig. 3:**
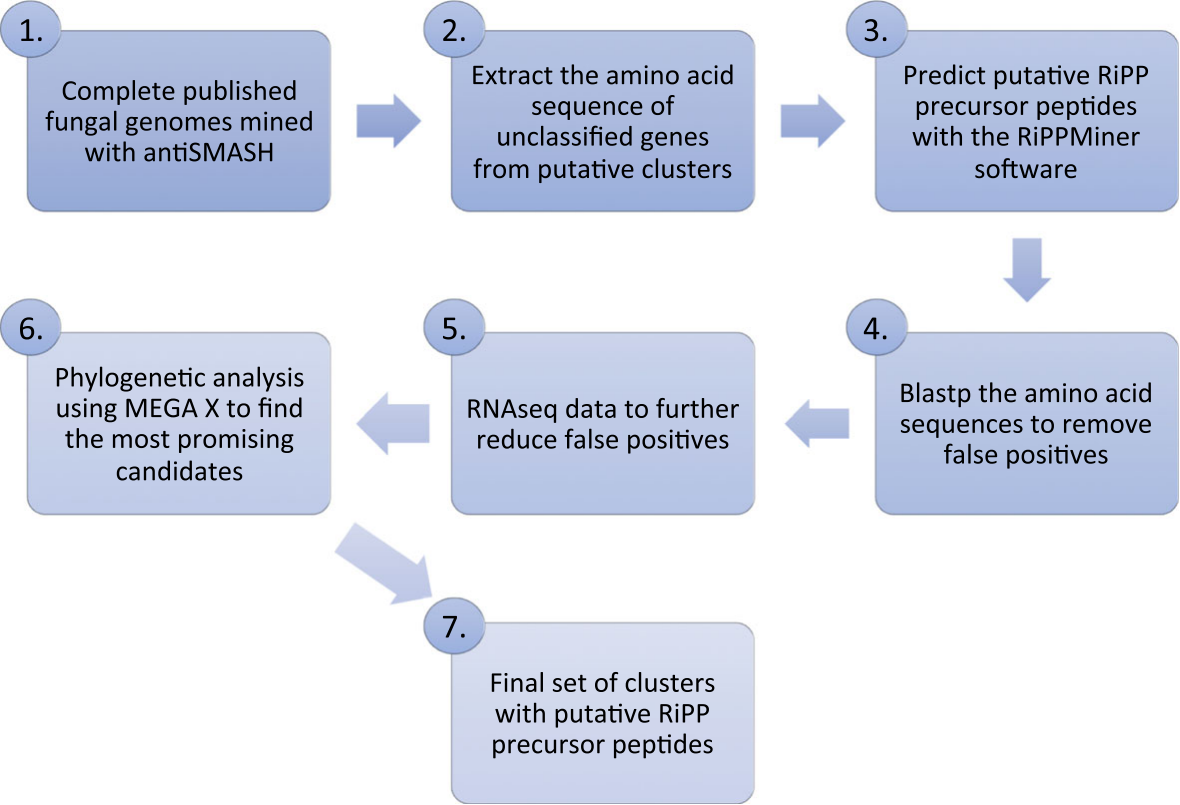
Schematic diagram of the pipeline used to discover possible RiPP precursor peptides

As we could verify the in principal applicability of the RiPPMiner for the identification of fungal RiPP precursors, we proceeded with the search for RiPPs in *Trichoderma* spp. As shown in Fig. 3, the amino acid query sequences were extracted from the results from antiSMASH ver. 4.3.0 from the “putative clusters” found by the ClusterFinder algorithm. Only genes without classification from antiSMASH were chosen as query sequences. This means that core biosynthetic genes, additional biosynthetic genes, transport-related genes and regulatory genes were not included in the RiPP prediction. The prediction was performed with the standalone version of RiPPMiner. The results of the RiPP mining procedure for the four *Trichoderma* spp. are shown in Table 2. For *T. harzianum* 23% of the query sequences were predicted to be putative precursor RiPPs. In the *T. reesei* genome 15% of the query sequences were recognized as putative precursor peptides by the RiPPMiner, for *T. citrinoviride* 17% of the query sequences and in the *T. brevicompactum* genome 22% of the query sequences were predicted as putative RiPP precursor peptides (Table 2).

**Table 2:** Number of predicted RiPPs (and subclasses) found by RippMiner

	***T. reesei***	***T. citirinoviride***	***T. harzianum***	***T. brevicompactum***
Query sequences	690	759	1099	518
Total predicted RiPPs	108	131	258	118
Cyanobactin	34	41	77	39
LanthipeptideB	10	7	18	4
LanthipeptideC	0	0	2	0
Lassopeptide	4	3	9	3
Linaridin	3	5	7	4
Microcin	1	5	5	0
Bacterial head to tail	0	0	1	2
Thiopeptide	1	0	0	0
Auto inducing peptide	0	1	0	0
NONE	54	69	140	66
After manual inspection	6	110	222	92

The protein sequences extracted from the predicted SM BGCs using antiSMASH ver. 4.3.0 (query sequences) were analyzed with the RiPPMiner software for *T. reesei*, *T. citrinoviride*, *T. harzianum*, and *T. brevicompactum*. False positives were removed via manual inspection

All amino acid sequences predicted to be RiPPs were manually inspected. This included aligning the sequences using Blastp v2.9.0+ [25] against the non-redundant protein database and a manually curated database of fungal proteomes to refine the search. Sequences with highly conserved active domains found in the Conserved Domain Database (CDD) [26] were removed, as well as classified sequences such as transcription factors, enzymes and ribosomal proteins. After manual inspection the sequences of *T. harzianum* were reduced to a final set of 222 sequences, *T. citrinoviride* was reduced to 110 and *T. brevicompactum* to 92. For *T. reesei* the genes for putative precursor sequences were furthermore compared to RNASeq data, and based on our analysis of the alignments to these genes, those without RNASeq data mapping to them were discarded as false positives. After further manual curation of the BGCs *T. reesei* was left with a final set of 6 putative RiPP precursor peptide genes.

### RiPP analysis by maximum likelihood method

**Fig. 4:**
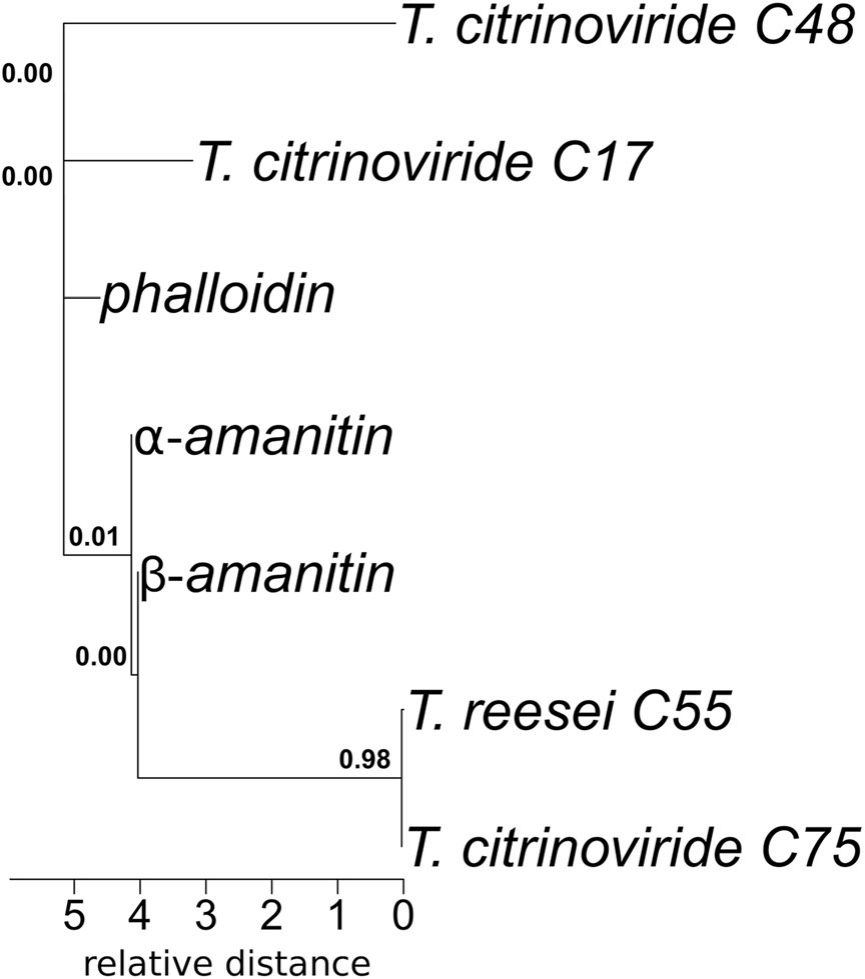
The extracted subtree showing the amanitin/phalloidin clade from a maximum likelihood phylogenetic tree. The ML phylogenetic tree was inferred based on 434 amino acid sequences. The evolutionary history was inferred by using the Maximum Likelihood method and Dayhoff w/freq. Model. The tree with the highest log likelihood (− 101,279.17) is shown. Initial tree(s) for the heuristic search were obtained automatically by applying Neighbor-Join and BioNJ algorithms to a matrix of pairwise distances estimated using a JTT model, and then selecting the topology with superior log likelihood value. The tree is drawn to scale, with branch lengths measured in the number of substitutions per site. The final dataset consisted of a total of 231 sites. The analyses were conducted in MEGA X [27].

We then inferred a maximum likelihood (ML) phylogenetic tree based on the putative precursor RiPP peptides from *T. reesei*, *T. citrinoviride*, *T. harzianum* and *T. brevicompactum*, the known fungal RiPP precursor peptides α-amanitin (A8W7M4), β-amanitin (ABW87785), phallacidin (ABW87771) and phalloidin (ABW87787), in order to find evolutionary linked sequences and to detect possible precursor peptide families. The analysis involved a total of 434 amino acid sequences, with sequence lengths ranging from 27 to 150 amino acids. Following the multiple sequence alignment computed with muscle [27], a total of 231 relevant positions were extracted for all sequences. These were used in the final data set to infer the phylogenetic distance corrected for multiple substitutions based on the substitution-rate matrices. The ML tree with the highest log likelihood (− 101,279.17) (Additional file 3) was used to extract the sub-trees including the putative RiPP precursor peptides from *T. reesei* and those including known precursor peptides of fungal RiPPs extracted from the UniProt database (Additional file 4). The branch lengths of the ML sub-tree are proportional to the relative distance between the sequences measured in the number of substitutions per site. As expected, the sequences of α-amanitin (A8W7M4), β-amanitin (ABW87785) and phalloidin (ABW87787) clustered together defining an own clade (Fig. 4). Within this amanitin/phalloidin sub-tree two sequences clustered closely together, one from the *T. reesei* set and one from *T. citrinoviride* (Additional file 1). These two putative RiPP precursor peptides were both defined by the RiPPMiner to be cyanobactins and have a bootstrap value of 0.98, making it highly likely that they are sisters to each other. Additionally, the *Trichoderma* sequences within this clade all showed high similarities to the putative structural toxin protein of *Eutypa lata* (UCREL1), the structural toxin protein RtxA of *Aspergillus oryzae* and *Metarhizium rileyi* in the Blastp output (Additional file 5).

**Fig. 5:**
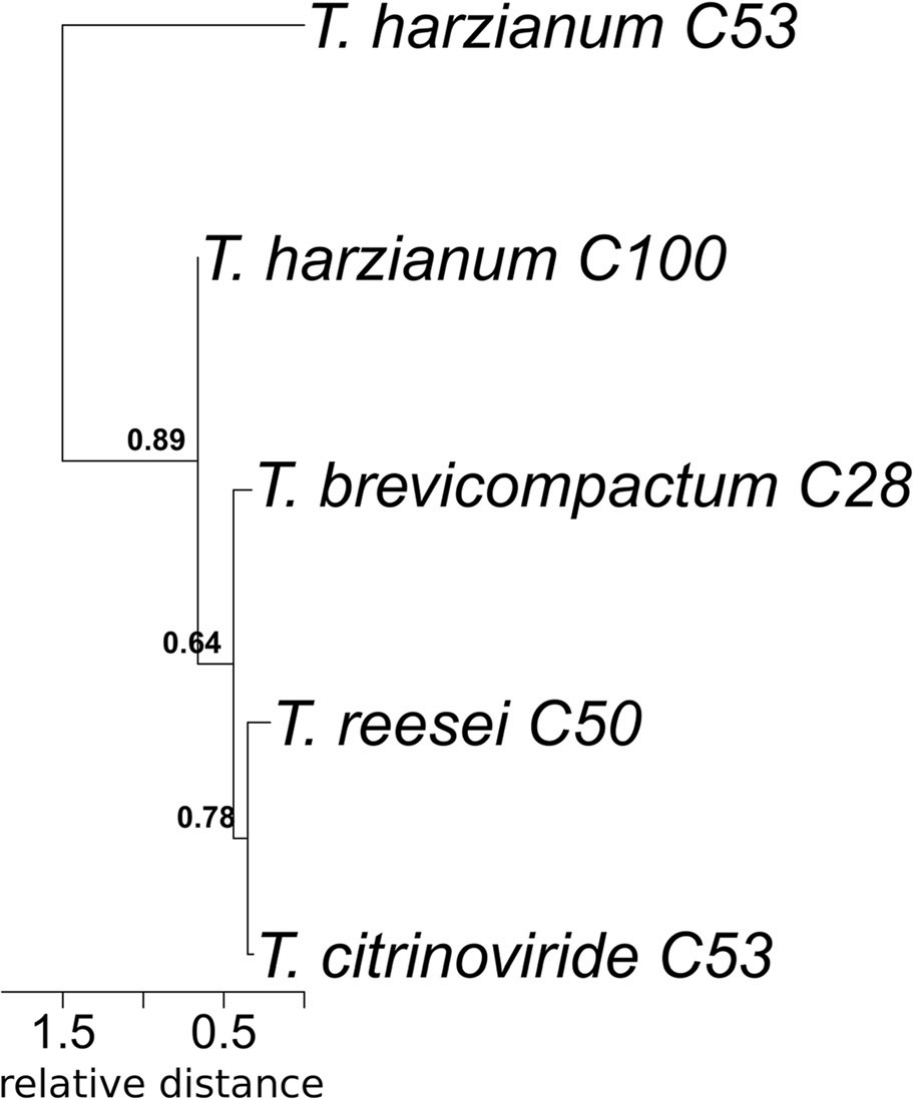
The extracted subtree showing the *Trichoderma-*putative-RiPP clade from a maximum likelihood phylogenetic tree. The ML phylogenetic tree was inferred based on 434 amino acid sequences. The evolutionary history was inferred by using the Maximum Likelihood method and Dayhoff w/freq. Model. The tree with the highest log likelihood (− 101,279.17) is shown. Initial tree(s) for the heuristic search were obtained automatically by applying Neighbor-Join and BioNJ algorithms to a matrix of pairwise distances estimated using a JTT model, and then selecting the topology with superior log likelihood value. The tree is drawn to scale, with branch lengths measured in the number of substitutions per site. The final dataset consisted of a total of 231 sites. The analyses were conducted in MEGA X [27].

We identified another outstandingly interesting subtree within the ML-tree. The amino acid sequence from *T. reesei* found in the BGCs 50 on contig 16 of the genome in the open reading frame 123 clusters within a conserved clade with the bootstrap values 0.64–0.89 (Fig. 5). The clade consists of one putative precursor RiPP peptide sequence from *T. harzianum*, *T. citrinoviride, T. brevicompactum* and *T. reesei* respectively. We called this clade the *Trichoderma-*putative-RiPP clade because the subtree of these sequences resembles the dendrogram in the heatmap, representing a whole genome phylogeny of the *Trichoderma* genus (Fig. 2). Within the other extracted sub-trees, the *T. reesei* putative RiPP precursor peptide sequences cluster with different sequences from each set of putative RiPP precursors namely *T. harzianum*, *T. citrinoviride* and *T. brevicompactum*. The sequences from the *T. harzianum*, *T. citrinoviride* and *T. brevicompactum* sets also made up own clades, these were not considered in the exploratory analysis due to the lack of RNASeq data for these specific strains and therefore the unknown high amount of false positive predicted RiPP precursor peptide sequences.

### Analysis of the putative RiPP cluster 55 of *T. reesei*

**Fig. 6:**
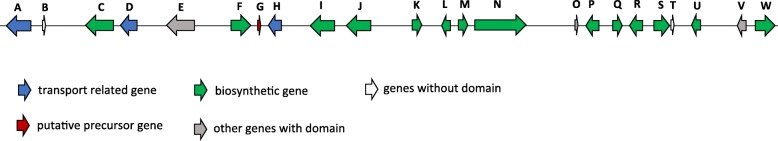
Schematic representation of biosynthetic gene cluster 55 of *T. reesei.* The gene cluster is located on scaffold 19 (571843–660,892 nt) and contains 22 predicted genes and two possible pseudogenes. Gene A is a putative general substrate transporter, position B is a possible pseudogene, gene C a glycosyltransferase from the family 1, gene D is a putative MFS general substrate transporter, gene E is a HET-domain-containing protein, gene F is a sulfatase, gene G is a putative RiPP precursor peptide, gene H is a putative amino acid transporter, gene I is a chitinase, gene J is a putative GMC oxidoreductase, gene K is a casein kinase II alpha subunit, gene L is a putative alpha/beta-hydrolase, gene M is a GroES-like protein, gene N is an acid phosphatase, gene O is a hypothetical protein, gene P encodes for a cytochrome P450, gene Q is a putative NAD (P)-binding protein, gene R is a family 54 glycoside hydrolase, gene S is a putative carboxypeptidase S, gene T is a possible pseudogene, gene U is a putative class I glutamine amidotransferase-like protein, gene V is a PTH11-type GPCR and gene W is a GMC oxidoreductase. The gene annotations were manually curated and based on a Blastp v2.9.0+ (protein-protein BLAST) [25] search against the non-redundant protein database, the conserved region finder and a manually curated database (Additional file 5)

Based on the phylogenetic and exploratory analyses of the putative RiPP precursor peptide sequences, we decided to perform a detailed analysis of a possible novel RiPP cluster found in *T. reesei*, namely cluster 55. Cluster 55 contains the putative RiPP precursor peptide that clustered in the ML tree in the amanitin/phalloidin clade (Fig. 4). This putative RiPP precursor peptide from *T. reesei* has a sister in *T. citrinoviride* with a high bootstrap value (Fig. 4). Furthermore, the RNASeq data showed that the putative RiPP precursor peptide from cluster 55 is transcribed at low levels. These findings highly suggest that this putative RiPP precursor peptide is indeed present in the genome of *T. reesei*. First, we manually annotated all genes in cluster 55 (as predicted by antiSMASH) by performing a Blastp v2.9.0+ [25] search against the non-redundant protein database, the conserved region finder and a manually curated database (Additional file 5). The results are visualized in Fig. 6. Gene D was classified as a putative major facilitator superfamily (MFS) general substrate transporter. Notably, the same kind of transporter is found in the ustiloxin B cluster of *A. flavus* [17]. Adjacent to the gene of the putative RiPP precursor peptide a sulfatase gene is encoded (Gene F). Additionally, a putative hydrolase (Gene L), acid phosphatase (Gene N), cytochrome P450 (Gene P) and peptidases (Gene S) are found. This gives the cluster 55 the potential arsenal of enzymes needed for posttranslational modification and transport of the finished putative RiPP. Notably, these and further genes of *T. reesei cluster* 55 have homologs in *T. citrinoviride* cluster 75 (Additional files 6, 7 and 8).

### RiPP precursor peptide analysis

**Fig. 7:**
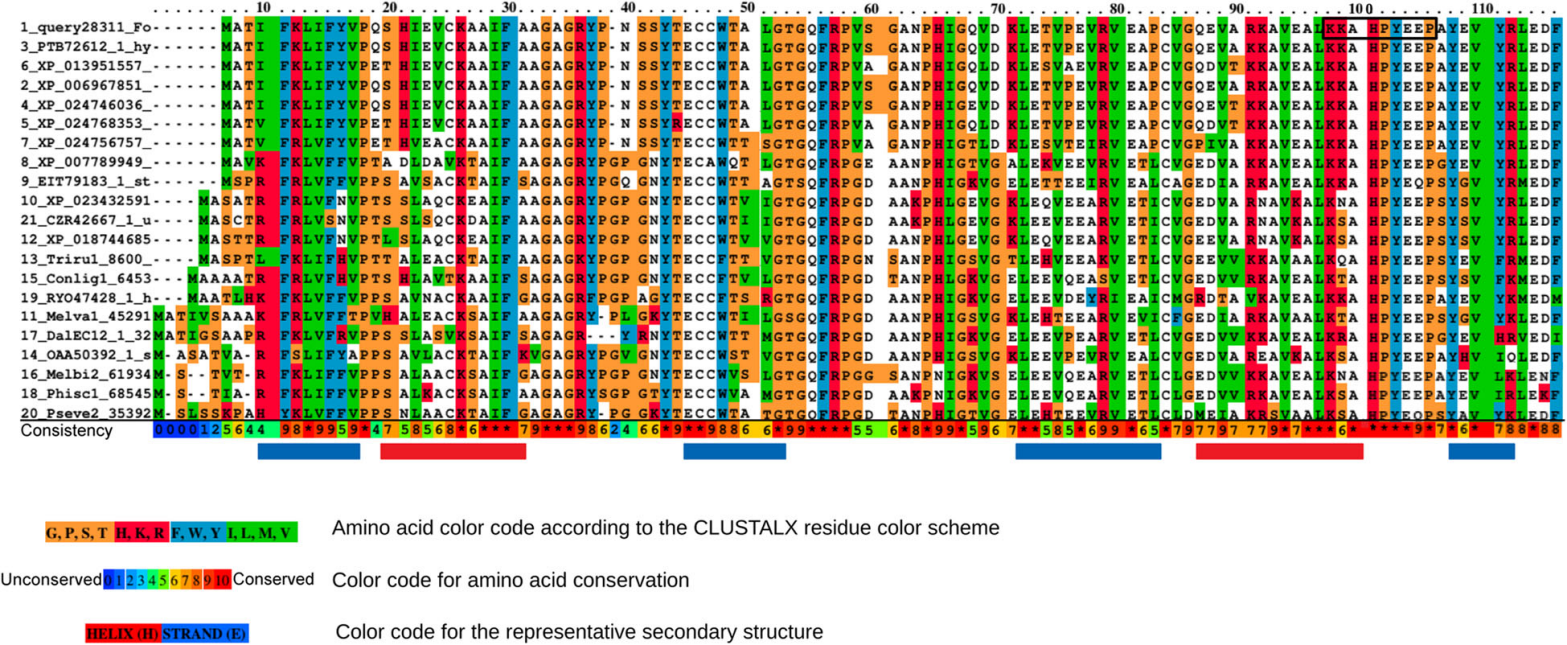
Multiple sequence alignment of the putative RiPP precursor peptide from cluster 55. The putative RiPP precursor peptide from *T. reesei* was aligned to the 20 top hits from the Blastp output using the ClustalW algorithm and was performed with PRALINE [28]. The 21 aligned amino acid sequences are colored according to the ClustalX residue color-scheme. The row labeled ‘Consistency’ is color-coded based on the amino acid conservation performed by PRALINE, 0 representing the least conserved alignment position colored in blue up to 10 marked by an asterix in red. Below the Consistency row the blue and red colored blocks stand for the representative secondary structure prediction using DSSP and PSIPRED. The *β*-Strand predictions are colored in blue and the red colored blocks are the *α*-Helix predictions. The predicted putative core peptide sequence is indicated by a black frame

Next, we performed a more in-depth analysis of the putative RiPP precursor peptide sequence found in cluster 55 of *T. reesei* (Fig. 6). The analysis involved a multiple sequence alignment with the 20 top hits from the Blastp output using the ClustalW algorithm and was performed with PRALINE [28] (Fig. 7). The putative RiPP precursor peptide from *T. reesei* is 109 amino acids long. There was no O-glycosylation potential predicted with NetOGlyc4.0 [29] and only a single low potential N-glycosylation site could be detected at the asparagine in position 33 with NetNGlyc1.0 [30]. There was no N-myristoylation site found nor was there a C-terminus appropriate for peroxisomal import detected. To detect DNA motif binding sites NsitePred [31] was used, only low probability motifs were found (below 0.264) not giving the precursor peptide the ability to bind DNA effectively. These analyses were performed to exclude the possibility of the putative precursor peptide being involved in transcriptional regulation. To determine the core sequence of the putative precursor peptide firstly the possible posttranslational modifications based on the enzymes found in the cluster 55 were evaluated. The adjacent sulfatase gene strongly suggests a sulfated residue. To detect an appropriate sulfating site within the peptide the Sulfinator [32] application was used. It found the Tyrosine in position 96 to be the only possible sulfated site within the peptide. This suggests residue 96 to lie within the core sequence. The RiPPMiner software predicted the core sequence to be residues 91 to 99, comprising the core sequence KKAHPYEEP (Fig. 7). The start of this putative core sequence is a typical peptidase cut site (KK) only found once in the putative precursor peptide. Tyrosine 101 (2 residues after the C-terminal end of the predicted core sequence) is a predicted phosphorylation site according to NetPhos3.1 [33]. This might suggest a possible activation site for further processing of the core peptide sequence. Furthermore, the predicted core sequence is highly conserved, the main part is not predicted to be part of either an alpha-helix nor a beta-sheet, and the amino acids of the possible predicted core sequence are mainly hydrophilic (Fig. 7), making them easily accessible for enzymatic posttranslational modification. These findings further support the peptide from residues 91 to 99 to be the core sequence or at least part of the core of the putative RiPP precursor peptide.

## Discussion

Using antiSMASH ver. 5.0 [16] for the search of BGCs returned one identified fungal RiPP cluster in the genome of *A. flavus*. This was expected since the underlying search for fungal RiPP clusters in antiSMASH ver. 5.0 [16] is based on the ustiloxin B cluster from *A. flavus*. In contrast, the search for BGCs in the *Trichoderma* spp. and the *A. phalloides* genomes yielded the same results using the last two versions of antiSMASH (ver. 4.3.0 [22] and ver. 5.0 [16]) and no predictions of fungal RiPP clusters. Our unconventional approach found a total of 615 potential RiPP precursor peptides in the 4 mined *Trichoderma* genomes. Notably, the results from our approach were obtained by using tools designed for bacterial sequences. This procedure would strongly benefit from a database of fungal RiPPs that could be integrated in the RiPPMiner software. Consequently, these findings have to be carefully manually inspected and thereafter verified by RNA sequencing data to reduce false positives, as we did for the *T. reesei* results in this study. As we have shown for *T. reesei* after careful inspection of the results we could reduce our set of potential RiPP precursor peptides from 108 to 6.

One of these predicted putative RiPP precursor peptides is found in the *Trichoderma-*putative-RiPP clade, suggesting the existence of a conserved putative RiPP precursor peptide within the *Trichoderma* genus. Another putative novel fungal RiPP cluster in the *T. reesei* genome is cluster 55 (Fig. 6). Its precursor peptide sequence clustered in the amanitin/phalloidin clade together with a sequence from *T. citrinoviride*. Furthermore, the putative precursor peptide sequences within this clade all showed high similarities to the putative structural toxin protein of *E. lata* (UCREL1), the structural toxin protein RtxA of *A. oryzae* and structural toxin protein RtxA of *M. rileyi* in the Blastp output. The putative precursor peptide found in this cluster shows in the potential core sequence a predicted sulfatation site similar to the one found in the known fungal RiPP precursor peptide α-amanitin. (Fig. 7) Our results largely support the hypothesis that fungal genomes contain biosynthetic gene clusters for RiPPs that might be a vast untapped source for possible new lead compounds with yet undescribed potential applications. Further in vitro and in vivo investigations are needed to be able to predict a preliminary biosynthetic pathway for the described RiPP clusters and to definitively classify these six clusters found in silico as novel fungal RiPP clusters in *T. reesei*.

## Conclusion

In this study we describe a novel, unconventional mining approach for the search for RiPPs in fungi. While this method offers new possibilities it also demands a rather long hands on time to refine the search, due to the lack of automatization. However, we could successfully find previously known fungal RiPPs and predict several putative novel RiPPs in the genus *Trichoderma*.

In the fight against the rising threat of multiresistant pathogenic strains, fungal RiPPs represent an indispensable new armament of possible diverse lead compounds. Our study is the first report of the potential of *Trichoderma* to produce RiPPs and might pave the way for further studies on fungal RiPPs in *Trichoderma*. The method described in our study will lead to further mining efforts in all subdivisions of the fungal kingdom.

## Methods

### Extraction of RNA and sequencing

The RNASeq data used in this study was generated in a previous study by Derntl et al. [23]. Therein the wild-type like *T. reesei* strain QM6a Δ*tmus53* strain [34] cultivated in Mandels-Andreotti medium [35] containing 1% carboxy methyl cellulose as carbon source. After 48 h of solid-state incubation at 30 °C, the RNA was isolated using the RNeasy Plant Mini Kit (Quiagen) and libraries were prepared using a TruSeq Stranded mRNA Sample Prep Kit including poly (A) enrichment (Illumina). The libraries were sequenced on a NextSeq500 instrument (Illumina) with paired-end 75 nt long reads [23].

### Full genomes

The genomes of *T. asperellum* CBS 433.97 (assembly Trias v. 1.0; BioSample accession: SAMN00769595), *T. virens* Gv29–8 (assembly TRIVI v2.0; BioSample accession: SAMN02744059), *T. arundinaceum* (assembly Trichoderma_arundinaceum_IBT40837_contigs; BioSample accession: SAMN06320351), *T. reesei* QM6a (assembly v2.0; BioSample accession: SAMN02746107), *T. citrinoviride* (assembly Trici v4.0; BioSample accession: SAMN05369575), *T. harzianum* CSB 226.95 (assembly Triha v1.0; BioSample accession: SAMN00761861), *T. atrobrunneum* (assembly ASM343991v1; BioSample accession: SAMN08325511), *T. brevicompactum* (assembly Trichoderma_brevicompactum_IBT40841_contigs; BioSample accession: SAMN06320626) and *T. koningii* (assembly JCM_1883_assembly_v001; BioSample accession: SAMD00028335) were downloaded from the NCBI database. Furthermore, the genomes of *Amanita phalloides* (assembly ASM198338v1; BioSample accession: SAMN05444494) and *Aspergillus flavus* NRRL3357 (assembly JCVI-afl1-v2.0; BioSample accession: SAMN05591370) were downloaded from the NCBI database, to evaluate our mining procedure.

### Genome mining

The command line version of antiSMASH ver. 4.3.0 [22] was used to mine the selected genomes for secondary metabolite biosynthetic gene clusters with following specifications in order to yield the best results for the fungal genomes. The taxon was specified with the option --taxon to be of fungal origin, the --clusterblast, −-subclusterblast and --knownclusterblast options were used to compare the identified clusters against a database of antiSMASH-predicted clusters, known subclusters that synthesize precursors and known gene clusters from the MIBiG database [36] respectively. The --smcogs option enables a search for BGCs of orthologous SM groups. Furthermore, the ClusterFinder algorithm was activated with the --inclusive option for additive cluster discovery. In parallel a genome wide HMMer analysis was performed by specifying the --full-hmmer option and the active site finder module with the --asf option. The results for *T. reesei*, *A. flavus* and *A. phalloides* were then cross referenced with the online version of antiSMASH ver. 5.0 [16] that includes the identification of fungal RiPP clusters.

To verify the presence of similar precursor peptides within the *Trichoderma* genus four full genomes were chosen based on their average nucleotide identity (ANI) calculated with a fast alignment-free implementation for computing whole-genome ANI between genomes called fastANI [37]. The choice which *Trichoderma* spp. were to be mined for RiPPs was based on their average nucleotide identity (ANI). Within the putative clusters, when applying an 85% ANI cutoff, of the chosen genomes the amino acid sequences of the genes classified as “other genes” were extracted and concatenated in a single file. Core biosynthetic genes, additional biosynthetic genes, transport-related genes and regulatory genes were not included. The extracted sequences were then analyzed using the standalone version of RiPPMiner [24] to predict possible RiPPs within the genomes. The method of RiPP prediction was tested on known precursor peptides of fungal RiPPs extracted from the UniProt database, namely α-amanitin (A8W7M4), β-amanitin (ABW87785), phallacidin (ABW87771) and phalloidin (ABW87787) and 75 diverse known precursor peptides (Additional file 1).

All extracted amino acid sequences, that were predicted as putative RiPP precursor peptides by the RiPPMiner software, were blasted using Blastp v2.9.0+ (protein-protein BLAST) [25] against the non-redundant protein database (All non-redundant GenBank CDS translations, PDB, SwissProt, PIR, PRF excluding environmental samples from WGS projects) to refine the search and a manually curated database (e.g. KEPs could be identified and removed). Sequences with highly conserved active domains were removed from the total set, as well as classified sequences such as transcription factors, enzymes and ribosomal proteins. Only hypothetical proteins, small secreted cysteine rich proteins of unknown function (SSCRP) and sequences without considerable similarities were kept. The refined putative RiPP precursor peptides and the known precursor peptides of fungal RiPPs as reference were aligned with MUSCLE and a Nearest-Neighbor-Interchange (NNI) tree with 100 Bootstraps using the Jones-Taylor-Thornton (JTT) model was inferred by using the maximum likelihood method and Dayhoffw/freq. Model. The analysis was conducted with the MEGA X software platform [27].

Further analysis, visualizations and exploratory data analysis were carried out in R v3.6.0 [38] with the following packages: phangorn v2.5.4 [39]; ape v5.3 [40]; ggplot2 v3.1.1 [41]; ggtree v1.17.1 [42]; gplots v3.0.1.1 [43]; stats v3.6.0.

### Transcriptome analysis

The raw RNASeq paired-end reads were aligned to the *Trichoderma reesei* QM6a genome (assembly v2.0; BioSample accession: SAMN02746107) without using predefined annotations. This was done following the protocol for mapping RNASeq reads with a 2-pass procedure described by Dobin and Gingeras with the software STAR v 2.7.0c [44]. The alignments were visualized with IGV v2.5.3 (Integrative Genomics Viewer) [45]. This procedure was chosen to reduce false positive putative precursor peptide gene calls. Putative precursor peptide genes to which RNASeq data aligned were considered true positives. A schematic diagram depicting the overall scheme of the pipeline used to discover and curate possible RiPP precursor peptides is illustrated in Fig. 3.

### RiPP precursor peptide analysis

The most highly likely putative RiPP precursor peptide from *T. reesei* was aligned to the 20 top hits from the Blastp output using the ClustalW algorithm and was performed with PRALINE [28]. Furthermore, the peptide sequence was analyzed with NetOGlyc 4.0 [29] to predict O glycosylation sites, NetNGlyc 1.0 [30] to find possible N glycosylation sites, NsitePred [31] to evaluate if there are probable DNA motif binding sites, NMT [46] was used to recognize glycine N-myristoylation sites of fungi and to detect if the C-terminus is appropriate for peroxisomal import, NetPhos 3.1 [33] to predict phosphorylation sites and ExPASy – Sulfinator [32] to find appropriate sulfatation sites within the peptide. Furthermore, the conservation scoring was performed with PRALINE and the secondary structure prediction was performed using the Define Secondary Structure of Proteins (DSSP) algorithm and PSI-blast based secondary structure PREDiction (PSIPRED).

## Supplementary information

**Additional file 1.** The output of the RiPPMiner prediction for known precursor peptides of fungal RiPPs extracted from the UniProt database.

**Additional file 2.** The output of the RiPPMiner prediction for the Ustiloxin B cluster extracted from the antiSMASH output.

**Additional file 3.** Full maximum likelihood (ML) phylogenetic tree. The ML phylogenetic tree was inferred based on 434 amino acid sequences.

**Additional file 4. **The extracted sub-trees including the putative RiPP precursor peptides from *T. reesei* and those including known precursor peptides of fungal RiPPs extracted from the UniProt database.

**Additional file 5.** Blastp v2.9.0+ output for Cluster 55, containing the predicted RiPP precursors with similarities to amatoxins.

**Additional file 6. **Comparison of biosynthetic gene cluster 55 of *T. reesei* and biosynthetic gene cluster 75 of *T. citrinoviride.*

**Additional file 7. **Blastp v2.9.0+ output for cluster 75 of *T. citrinoviride*, containing the predicted RiPP precursor.

**Additional file 8. **Gene annotations for cluster 75 of *T. citrinoviride*, manually curated and based on a Blastp v2.9.0+ (protein-protein BLAST) [25] search against a manually curated database, including protein accessions.

## Data Availability

The datasets analyzed during the current study are publicly available in the NCBI National Center for Biotechnology Information repository, https://www.ncbi.nlm.nih.gov/genome/browse/#!/eukaryotes/. The datasets supporting the conclusions of this article are included within the article and its additional files.

## Abbreviations

ANI: Average nucleotide identity
BGC: Biosynthetic gene cluster
DSSP: Define Secondary Structure of Proteins
JTT: Jones-Taylor-Thornton
KEP: KEX2-processed repeat proteins
KEX2: Fungal Kexin-like proteinases (killer expression)
ML: Maximum likelihood
NNI: Nearest-Neighbor-Interchange
NRPS: Non-ribosomal peptide synthetase
PSIPRED: PSI-blast based secondary structure PREDiction
RiPPs: Ribosomally synthesized and post-translationally modified peptides
SM: Secondary metabolite
SSCRP: Small secreted cysteine rich proteins
T1pks: Type I Polyketide synthase
