# Supplementary figures and images for: Novel approach in whole genome mining and transcriptome analysis reveal conserved RiPPs in Trichoderma spp
Source: BMC Genomics. 2020 Mar 27;21:258. doi: 10.1186/s12864-020-6653-6 (PMC7099791; doi:10.1186/s12864-020-6653-6)

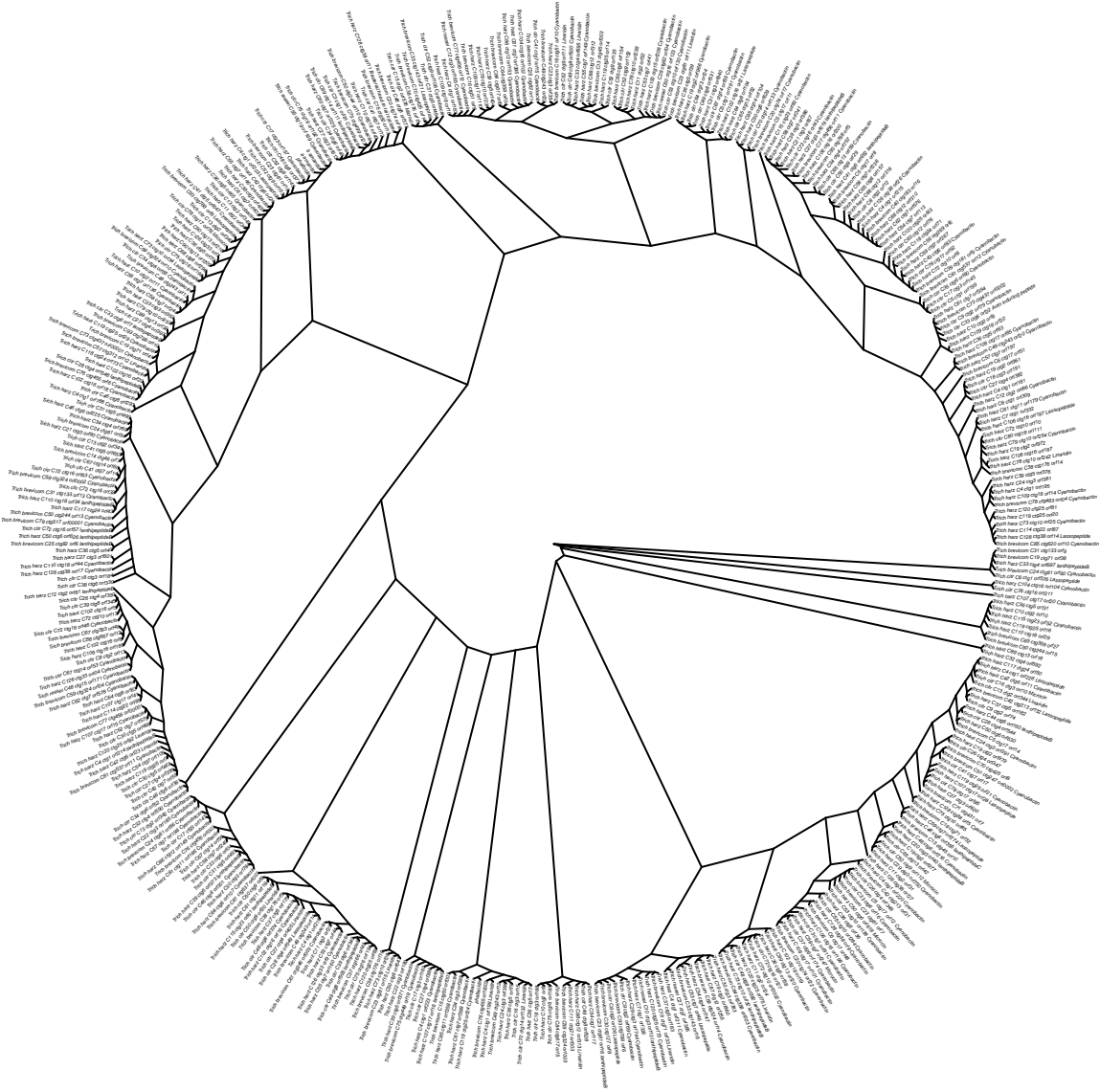

Supplement: Supplementary file 3 — Additional file 3. Full maximum likelihood (ML) phylogenetic tree. The ML phylogenetic tree was inferred based on 434 amino acid sequences. [file 12864_2020_6653_MOESM3_ESM.pdf]

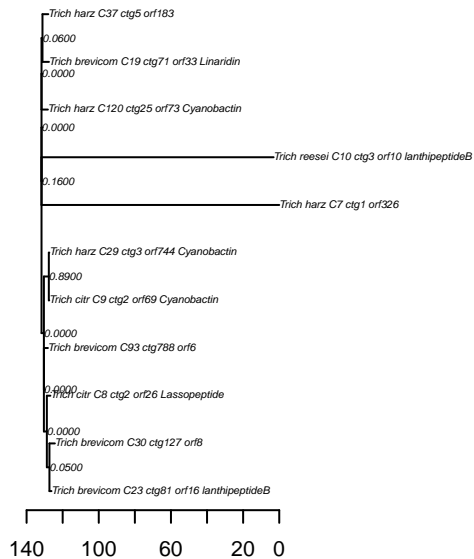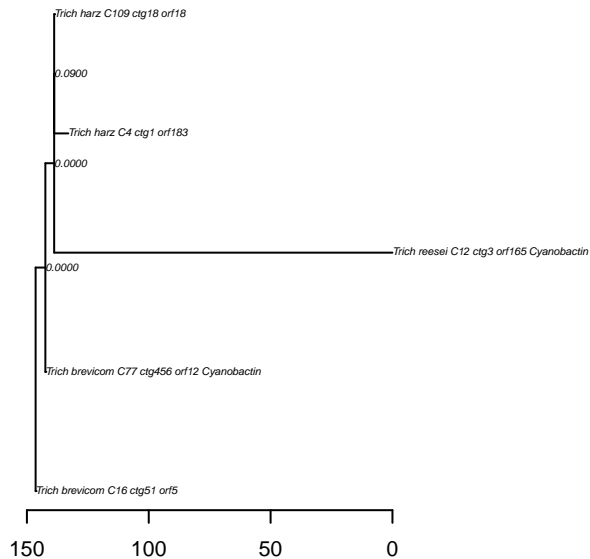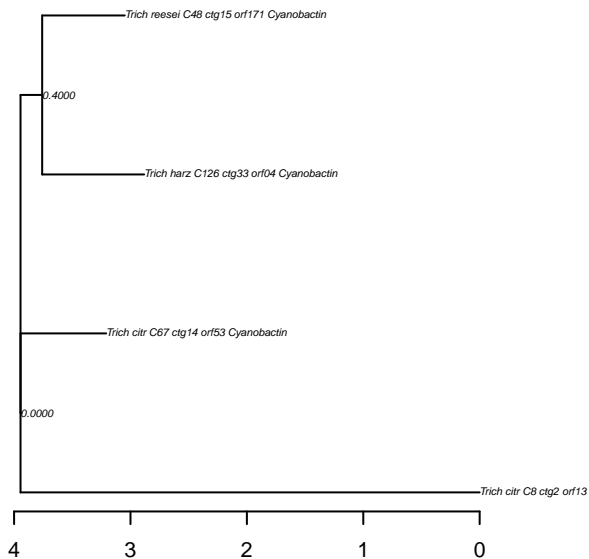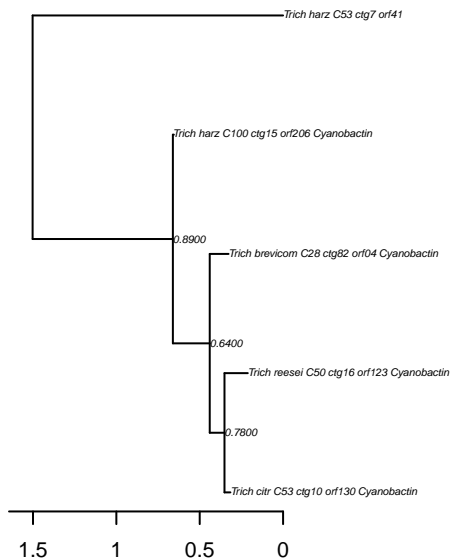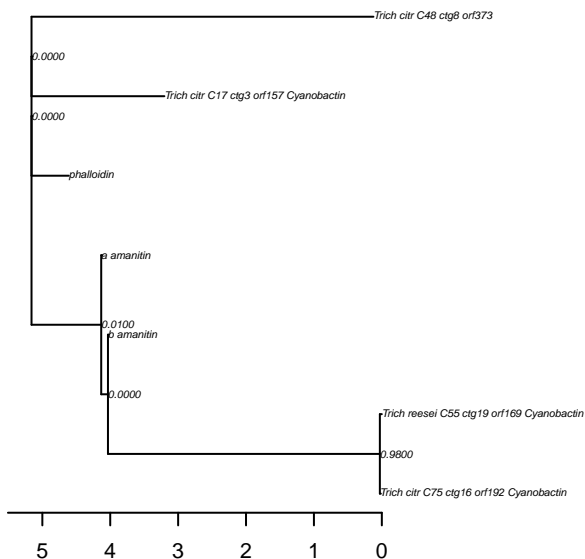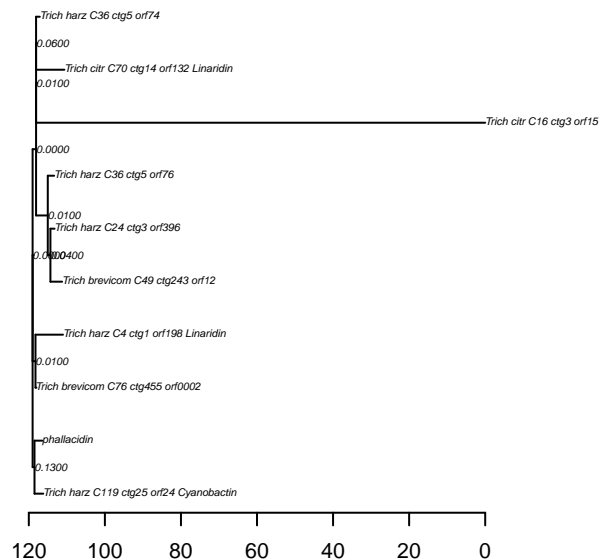

Supplement: Supplementary file 4 — Additional file 4. The extracted sub-trees including the putative RiPP precursor peptides from T. reesei and those including known precursor peptides of fungal RiPPs extracted from the UniProt database. [file 12864_2020_6653_MOESM4_ESM.pdf]
