# Supplementary material for: Novel approach in whole genome mining and transcriptome analysis reveal conserved RiPPs in Trichoderma spp
Source: BMC Genomics. 2020 Mar 27;21:258. doi: 10.1186/s12864-020-6653-6 (PMC7099791; doi:10.1186/s12864-020-6653-6)
Supplement: Supplementary file 6 — Additional file 6. Comparison of biosynthetic gene cluster 55 of T. reesei and biosynthetic gene cluster 75 of T. citrinoviride. [file 12864_2020_6653_MOESM6_ESM.pdf]

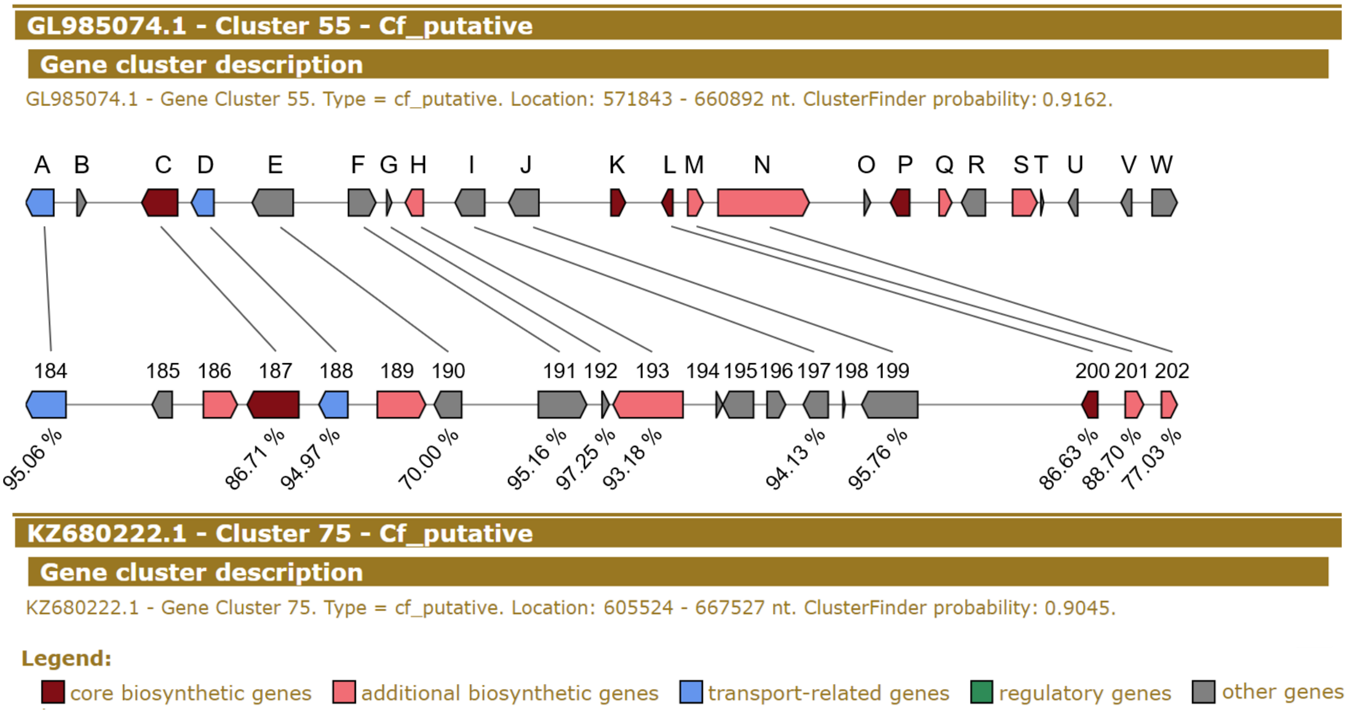

Additional file 6. Comparison of biosynthetic gene cluster 55 of *T. reesei* and biosynthetic gene cluster 75 of *T. citrinoviride*. The gene cluster of *T. citrinoviride* is located on scaffold KZ680222.1 (605524-667527 nt) and contains 16 predicted genes and three possible pseudogenes. The schematic representation of the two clusters was extracted directly from the antiSMASH results. The gene annotations were manually curated and based on a Blastp v2.9.0+ (protein-protein BLAST) (25) search against a manually curated database (Figure 6, Additional file 7). The gene designations of the *T. reesei* cluster 55 genes is the same as in Figure 6. The number of the open reading frames (orf) assigned by antiSMASH in the *T. citrinoviride* cluster 75 begin are indicated above the genes. All annotations and protein accession numbers for their corresponding orf can be found in Additional file 8. Orf 184 encodes a general substrate transporter, orf 185 a possible pseudogene, orf 186 a aldehyde dehydrogenase, orf 187 a glycosyltransferase family 1 protein, orf 188 encodes for a major facilitator superfamily (MFS) general substrate transporter, orf 189 a carbon-nitrogen hydrolase, orf 190 encodes for a Heterokaryon incompatibility protein, orf 191 a sulfatase, orf 192 the putative RiPP precursor peptide with the Location 636556 – 636948 nt, orf 193 a amino acid transporter, orf 194 a possible pseudogene, orf 195 a hypothetical protein, orf 196 a putative fungal transcription protein, orf 197 a carbohydrate-binding module family 1 protein, orf 198 a possible pseudogene, orf 199 a GMC oxidoreductase, orf 200 a alpha/beta-hydrolase, orf 201 a GroES-like protein and orf 202 encodes for a putative 3-hydroxyisobutyrate dehydrogenase.

The lines between genes of the two clusters indicate homology. The percentages beneath the *T. citrinoviride* genes represent the sequence similarities between the two homologous genes. Genes O – S, U W have homologs in *T. citrinoviride* (Additional file 5) at the corresponding location, but this is not depicted here, because antiSMASH did not predict these genes to be part of the BGC in *T. citrinoviride*.
